# Use of Telemedicine to Improve Neonatal Resuscitation

**DOI:** 10.3390/children6040050

**Published:** 2019-04-01

**Authors:** Lee T. Donohue, Kristin R. Hoffman, James P. Marcin

**Affiliations:** University of California at Davis Children’s Hospital, 2516 Stockton Blvd, Sacramento, CA 95817, USA; krhoffman@ucdavis.edu (K.R.H.); jpmarcin@ucdavis.edu (J.P.M.)

**Keywords:** telemedicine, telesimulation, neonatal resuscitation

## Abstract

Most newborn infants do well at birth; however, some require immediate attention by a team with advanced resuscitation skills. Providers at rural or community hospitals do not have as much opportunity for practice of their resuscitation skills as providers at larger centers and are, therefore, often unable to provide the high level of care needed in an emergency. Education through telemedicine can bring additional training opportunities to these rural sites in a low-resource model in order to better prepare them for advanced neonatal resuscitation. Telemedicine also offers the opportunity to immediately bring a more experienced team to newborns to provide support or even lead the resuscitation. Telemedicine can also be used to train and assist in the performance of emergent procedures occasionally required during a neonatal resuscitation including airway management, needle thoracentesis, and umbilical line placement. Telemedicine can provide unique opportunities to significantly increase the quality of neonatal resuscitation and stabilization in rural or community hospitals.

## 1. Introduction

Approximately 5% of babies born at term gestation require additional resuscitation beyond drying and stimulation to initiate spontaneous respirations. While many of these newborns respond to effective positive pressure ventilation, approximately 2% require intubation with others requiring more extensive resuscitation including medication administration [1]. Although this percentage is small, the total number of births is large and babies that require more advanced resuscitation represent an important cohort of newborns that deserve special attention and innovative models of care. 

It is essential that the team responding to neonatal emergencies be well trained and able to expertly perform their respective tasks. This is important because the care provided within the first few minutes of life could play a major role in the reduction of neonatal morbidity and mortality. We know that neonatal resuscitation training improves neonatal and perinatal mortality [2]. The traditional method of neonatal resuscitation training, the Neonatal Resuscitation Program (NRP) from the American Academy of Pediatrics, is a single initial in-person course followed by another shorter in-person review course every two years. For staff at many smaller hospitals, this may be the only regular resuscitation training received in regard to the resuscitation of critically ill newborns. 

Neonatal death rates are higher at hospitals with a lower number of annual births [3]. This is likely related to the lack of neonatal resuscitation experience the staff at smaller delivery hospitals gain outside of NRP training. In these smaller hospitals, the low delivery rate and low frequency of deliveries that require advanced resuscitation make it difficult for staff to gain and maintain their resuscitation skills. One study of 26 rural hospitals demonstrated that neonatal resuscitation skills had not been performed in the last year by many providers and there was a correlation between frequency of skill performance and performance comfort level [4]. In these settings, practitioners have few opportunities to use the knowledge and skills learned in the NRP training, which leads to knowledge decay and difficulty in maintaining requisite skills. 

In hospitals without neonatal intensive care units (NICUs), the ability to effectively perform newborn resuscitation and stabilize sick and preterm infants until transfer is imperative to best care practices. The mortality and morbidity rates for premature and acutely ill newborns that require transport are higher than for those born at hospitals with a NICU [5]. For very low birth weight and very preterm infants, birth outside of a level III hospital is significantly associated with increased likelihood of death [6]. Mortality rates among very low birthweight (VLBW) infants are lowest for deliveries that occurred in hospitals with higher level NICUs and a high volume of such patients [7]. It is likely that some of the difference in outcomes is related to the increased frequency of skill use and training in NICUs.

Unfortunately, neonatal intensive care units and neonatologists are highly regionalized, leaving large areas without immediate access to neonatal expertise and neonatal intensive care resources [8,9,10,11]. Urban areas contain 99% of NICUs in the United States [11]. This results in significant regional variation in the availability of neonatal and perinatal care for women. In a recent study, although 93% of women of reproductive age in the United States live within 50 miles of a NICU, 21 states had less than 80% living with 50 miles of both an obstetric critical care unit and NICU and three states had less than 20% living within 50 miles of both of these services [11]. In California, of the 255 hospitals with recorded births in 2016, only 124 (49%) have NICUs of any level [12]. Though many high-risk infants can deliver in hospitals with a NICU, some deliveries happen too quickly to transfer mothers to a higher level of care or otherwise routine deliveries that may involve unexpected complications or unanticipated infant conditions. The large number of community and rural hospitals without NICUs but with delivery services causes many infants to deliver in a hospital where the resuscitation team has a lack of experience and training in neonatal resuscitation.

Telemedicine, or the delivery of care over a distance using telecommunication technologies, has numerous applications in neonatology. Several papers describing the use of telemedicine in pediatrics and neonatology have recently been published. There have been publications from the American Academy of Pediatrics reviewing the applications of telemedicine [13] as well as a policy statement on how telemedicine can be used to address access and workforce shortages [14]. This technical report and policy statement were published in 2015 and have been followed by publications documenting the expanding role of telemedicine in the care delivery of children. In 2018, Olson, et al., published a survey-based summary of more than 50 programs in the US representing 30 states that provided data on implementation, barriers, staffing resources, operational processes, technology and funding sources used in their telehealth programs [15]. Sauers-Ford, et al. published a review of the uses of telemedicine to address disparities in access to specialist care for neonatal patients in 2018 [16]. Most recently, Hoffman, et al. published a review of Telemedicine in Neonatology which provided a historical perspective of telemedicine and briefly discusses all of the clinical applications of telemedicine in neonatology [17].

We should note that despite the increasing excitement, use and published research on the use of telemedicine, there remain important limitations to the use of telemedicine. Telemedicine requires some technology to be present at both communicating sites. This technology must be readily available to the remote specialist who may not be in a hospital and to the patients who may be in a variety of environments, such as an emergency department, a newborn nursery, or an operating or delivery room. The technology also requires an initial capital and personnel investment by the hospital system. The cost of video-conferencing equipment can range from simple integrated cameras or webcams that operate using existing software to turnkey, high-definition systems with peripheral devices such as stethoscopes. Similarly, telecommunications can be as simple as secure internet connections to dedicated, bridged networks that can ensure quality of service. Finally, personnel, must view telemedicine as beneficial to providers on both ends of the connection. There are many instances where telemedicine equipment sits unused due to lack of provider engagement. This may be compounded by the need for training and the resources and technical support to troubleshoot systems [15].

We will focus this review specifically on the use of telemedicine in neonatal resuscitation. Telemedicine is a relatively low-resource model of care that can be used to improve neonatal resuscitation and stabilization of newborns, particularly at rural and underserved labor and delivery units and has the potential to reduce morbidity and mortality in this group of patients. We will discuss various uses of telemedicine in neonatal resuscitation including education, training and debriefing, direct assistance of resuscitation, and support during critical procedures. Telemedicine is increasingly used to help bridge the gap between rural and community nurseries and tertiary NICUs in neonatal resuscitation outcomes and is expanding the reach of highly regionalized neonatologists. 

## 2. Neonatal Resuscitation Training

The traditional method of training neonatal resuscitation teams is with in-person training every 2 years for the maintenance of NRP certification. However, it is becoming clear that this alone may be insufficient to maintain resuscitation and communication skills. A review of 47 cases of perinatal death or permanent disability reported to the Joint Commission for review under the Sentinel Event Policy identified communication, staff competency and training as the top root causes [18]. The most recent International Liaison Committee on Resuscitation (ILCOR) resuscitation consensus statement further highlights this issue by presenting evidence that there is decay in skills and knowledge within months after initial training [19]. The paper also states that more frequent training is needed although the optimal frequency and method of training remains undetermined [19].

There have been several studies attempting to address the issue of the ideal frequency of training in neonatal resuscitation. There is a decline several months after training [20,21,22,23,24] and this seems to be more rapid for resuscitation skills than knowledge [23]. There is improvement after relatively brief simulation-based review sessions occurring 6–12 months after initial NRP training [22,24]. Brief, frequent manikin-based sessions were also recommended in the ILCOR consensus statement [19] and American Heart Association guidelines [25]. Education through telemedicine and telesimulation can be used to address these issues.

Increased frequency of training is particularly needed in rural and community hospitals. Staff at regional perinatal centers gain experience through their large volume of high-risk deliveries and often hold more regular resuscitation trainings. However, providers at small hospitals have more difficulty maintaining their resuscitation skills due to the low frequency of neonatal emergencies. Barriers to the frequent universal training of staff in responding to neonatal emergencies at more rural locales and community-based hospital settings include availability of resuscitation training experts and equipment and geographic constraints. It is resource-intensive to transport either the staff to the larger center with trainers and equipment or the trainers and simulation equipment to the remote site. 

Recent technological advances have led to the emergence of telesimulation, which combines the benefits of simulation-based teaching and remote video-conferencing to provide education, training, and assessment to off-site learners in a resource-effective manner. Telesimulation allows an experienced, trained simulation instructor to provide education to remote learners. This is particularly useful for rural providers who otherwise may have to spend a significant amount of time traveling to receive the same educational experience [26]. Conversely, telesimulation allows a trained simulation instructor to provide more training sessions when the time of time traveling to rural sites is eliminated. Providing education through telesimulation may also offer a way for larger centers to foster new relationships with remote hospitals, including the sharing of evidence-based algorithms, protocols and approaches to non-standardized care. These relationships are becoming more important in the current era of academic medical centers forming relationships with many affiliate sites [27].

The simplest form of telesimulation, teledebriefing, connects an expert to learners over a video-conferencing system to view a simulated scenario and guide post-resuscitation (real or simulated) education and discussion [28]. While this may seem unnecessary if the outside center has all of the simulation equipment, it is important to remember that reflection and learning are the greatest during the debriefing phase of simulation [29]. Debriefing should be performed by faculty members who have both expertise in debriefing as well as medical knowledge of the patient scenario. There is often a lack of these qualified experts at centers and particularly at smaller community sites. Debriefing over telemedicine offers an easy and economical way to bring these experts to the learners to optimize the educational benefit of simulation. 

**Figure 1:**
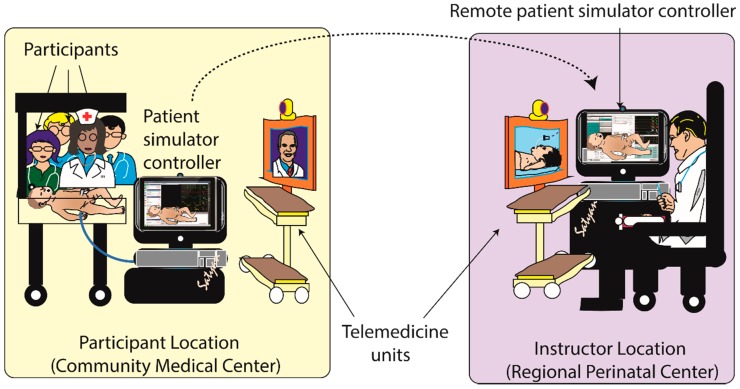
Remote telesimulation. Participants learning resuscitation using a neonatal patient simulator controlled at a remote site. A remote patient simulator controller PC connects to the patient simulator controller PC at that remote site that is directly connected to the patient simulator. An instructor at the remote site can view the simulated resuscitation and debrief with the participants. Copyright Satyan Lakshminrusimha, MD.

**Figure 2:**
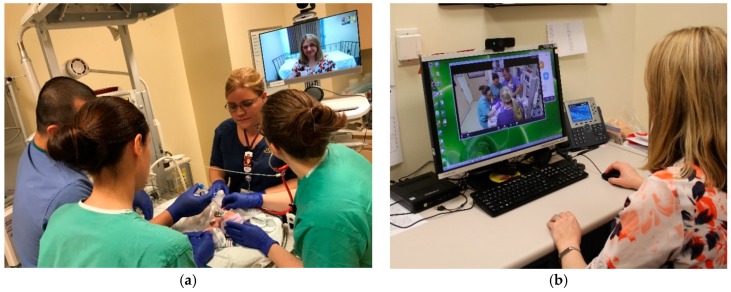
Telesimulation at University of California, Davis Children’s Hospital. (**a**) Remote participants resuscitating a neonatal patient simulator; (**b**) Instructor viewing the resuscitation over a telemedicine connection.

More sophisticated options include experts completely controlling a high-fidelity simulator and running a scenario/debrief much as you would if the learners and experts were at the same facility (Figure 1 and Figure 2). The feasibility of telesimulation has been described in a number of papers [26,30,31,32] including transoceanic telesimulation [33,34]. Multiple studies have also reported that the effectiveness of remote telesimulation is at least equal to in-person training [32,35,36,37], although one study reported communication issues between participants and the facilitator [36]. Telemedicine with telesimulation has been shown to be a useful method for teaching basic first aid [38] and Advanced Trauma Life Support [39]. In a study focused specifically on neonatal resuscitation training via telemedicine, nurses were randomized to either classroom teaching or tele-education of neonatal resuscitation by didactic lectures and demonstrations. The results showed similar improvement in both knowledge and skills in the two groups which suggests that tele-education may be an effective way to teach neonatal resuscitation [40]. In addition, off-site simulation experts can provide support to local simulation programs by assisting in the design of scenarios and technological set-up as well as providing feedback on performance and debriefing [41]. Together, these telemedicine strategies may expand the NRP training and maintenance of skills in rural and community nurseries. 

## 3. Neonatal Resuscitation

Telemedicine is also used for direct assistance during neonatal resuscitation. In this case, a remote critical care expert can connect via a video-conferencing method to be “virtually present” to provide direct assistance to the healthcare provider team at a remote site. This has been shown to be feasible in the adult [42,43,44], pediatric [45,46,47] and neonatal [48] populations. 

There are some studies that suggest remote assistance from an expert provider may be beneficial to patients. In one case series of patients presenting to remote sites with respiratory or cardiac arrest, the remote providers felt that the assistance they received via telehealth aided assessment, assisted communication and improved the quality of patient care [49]. In another study in which the remote pediatric intensivist communicated with pediatric residents, there was an improved quality of respiratory insufflations and chest compressions during a simulated patient emergency [50]. Telemedicine assistance provided during neonatal resuscitation and stabilization was shown to improve provider perception of teamwork as well as patient safety and quality of care [48]. Video-assisted resuscitation has also been shown to improve the time to effective ventilation including the improved use of corrective steps, decreased intubation and increased adherence to NRP in simulated resuscitation [51]. A recent retrospective study compared neonates that received telemedicine support during resuscitation to those that did not and showed that the telemedicine group had an improved resuscitation quality rating. The difference in resuscitation quality was more pronounced in the preterm subgroup and this subgroup was more likely to have important interventions such as temperature measurements and a blood gas sent [52].

In other applications, telemedicine has been shown to assist in the assessment of neonatal patients in distress. In one study, neonatologists evaluated infants with respiratory distress via a phone call only versus phone call with video. Their ratings of the stability of the patient were altered and their confidence in their assessment improved with the addition of video. They were also less likely to recommend intubation of infants with mild respiratory distress in the video group [53]. 

Numerous methods to provide resuscitation support via telemedicine are available. Generally, the minimum requirements for telemedicine include a camera and audio at each site with a secure telecommunications connection. The camera systems can be specifically developed for telemedicine use (turnkey systems) or can be universal cameras that are operated by local software. The cameras range from basic video webcams to more sophisticated cameras with optical and digital zoom and pan-tilt-zoom (PTZ) capabilities. The telemedicine cameras can be packaged on a cart, pole or wall or ceiling mounts. More recently, software and applications are being used on mobile devices including laptops, tablets, and cellular phones which can use integrated camera and audio. The diversity in options allows for hospitals and providers to find a suitable solution that meets their respective clinical needs. 

While there are many NICUs providing telemedicine support to remote nurseries, there is a lack of published data on the use of telemedicine to facilitate resuscitation. Our experience at UC Davis Children’s Hospital has been quite positive. We have convinced many referring providers that telemedicine is a useful tool in education and the delivery room. For more than 8 years, we have been providing training, emergency resuscitation assistance and non-urgent newborn nursery consultations via telemedicine to rural community labor and delivery units and nurseries. Telemedicine has allowed our experienced neonatologists the ability to provide on-demand bedside assistance to newborn nurseries across a broad geographical area. Neonatologists routinely provide guidance during high stress resuscitations, adding to the quality of care and potentially reducing the disparities between infants born in non-NICU community nurseries and tertiary referral NICUs. Importantly, telemedicine has also been able to provide reassurance and guidance to nurseries for non-critically ill infants, reducing the number of transfers to NICUs, reducing healthcare costs and avoiding the separation of the mother–baby dyad. 

Another novel use of telemedicine to improve the care of high-risk infants and their families born outside of a regional perinatal center is providing counseling to parents. This can either be done prior to the delivery if there is sufficient time or after delivery. This counseling is routinely provided to patients presenting to centers with perinatologists and neonatologists. However, when the delivery occurs unexpectedly at a smaller center or there is insufficient time to transport the mother prior to delivery, the family can receive inadequate information. We have successfully provided this service over our telemedicine network at UC Davis. Finally, telemedicine technologies can be used to deliver care internationally, particularly to resource limited countries/regions. While this topic is beyond the scope of this review, it is expected that as technologies become more affordable and telecommunications improve, bedside assistance to newborns in developing countries during resuscitation will become commonplace.

Our group also has experience with the use of telemedicine to guide the cessation of resuscitation. The guidelines now encourage discontinuation of resuscitative efforts if there has not been a heart rate for over 10 min [54]. However, making this decision is often an agonizing one for less experienced providers. A neonatologist viewing the resuscitation can help guide providers in the cessation of resuscitation when severe morbidity or mortality is likely. Our group has been able to counsel both providers and families when resuscitative efforts are felt to be futile. Providers are often thankful to have this decision made in conjunction with an expert. Families are more likely to feel that “everything” was done for their newborn in these extremely difficult situations when a neonatologist is guiding the resuscitation. While such outcomes are unusual in neonatal resuscitation, the virtual presence of a neonatologist is extremely valuable. 

## 4. Telemedicine Supported Procedures

Addressing another critical but uncommon skill for neonatal resuscitation, telemedicine can also be used for remote procedural training. Telemedicine technologies have been used to train physicians in intraosseous needle placement [55] and ultrasound-guided procedures [56]. There have also been reports of the use of telemedicine to train and supervise fetal, cardiac and vascular ultrasonography by remote sonographers [57]. 

Telemedicine can be used to aid remote physicians who may be required to perform emergency procedures. For example, a study in which medics performing thoracotomy tube placement in simulated patients were randomized to being remotely assisted or not showed an improvement in success from 71% in the unassisted group to 100% in the assisted group [58]. There have been several studies looking at the use of telemedicine to assist in airway management. Improvement was seen in intubation success as well as time to intubation of simulated patients by emergency medicine nurses [59]. There also is evidence that remote airway assistance is feasible in real patients using video laryngoscopy [60,61]. One study described 206 telemedicine-assisted intubations performed in rural emergency departments in which 71% were successful on the first attempt and 96% overall success despite most of these having at least one attempt prior to initiating the telemedicine consultation [62]. Another cross-over study in which medical students were randomized to four groups (telemedicine supervised direct laryngoscopy, in-person supervised direct laryngoscopy, telemedicine supervised video laryngoscopy and in-person supervised video laryngoscopy) demonstrated no difference between success rates in the groups which suggests that telemedicine supervision is at least as effective as in-person supervision [63]. 

The use of telemedicine to assist in video laryngoscopy for neonatal intubation is less commonly used, particularly in community hospitals, but this technology is available and could be used in the future to assist in airway management. The other neonatal procedures that are required during neonatal resuscitation and stabilization are needle thoracentesis and umbilical line placement. On several occasions, neonatologists at UC Davis have directed the placement of umbilical lines and performance of needle thoracentesis via telemedicine. Pediatric cardiologists have also assisted in the remote diagnosis of critical congenital heart disease, assisted in the initial management and stabilization, conducted remote cardioversion for supraventricular tachycardia, and even counseled parents regarding the immediate care and transfer plans for those infants that require immediate transfer to a regional NICU. Training in these procedures in telemedicine-led simulation sessions may also increase providers comfort and skill in these important procedures. 

## 5. The Future of Telemedicine in Neonatal Resuscitation

As telemedicine becomes more widely used, several new areas of telemedicine and telesimulation use may develop. Telemedicine support for endotracheal intubation using video laryngoscopy may ultimately lead to robotic assistance for neonatal intubation. Similarly, telemedicine guidance for umbilical line placement could be augmented by robotics. Telesimulation may be used as part of orientation or training for new nurses, respiratory therapists, or physicians with direct feedback and support from more experienced neonatal caregivers, allowing rapid dissemination of knowledge. 

However, telemedicine and telesimulation require further study in several areas prior to more broad use. While the use of the digital stethoscope and other digital tools in adult telemedicine is common, neonatal digital stethoscopes are not yet available. However, the development of a neonatal digital stethoscope may improve the ability of a neonatologist to provide assist rural/community providers. Similarly, improved internet speed and camera resolution may improve the telemedicine and telesimulation experience. The increased fidelity of manikins may allow more realistic training in umbilical line placement, endotracheal intubation, and needle thoracentesis. Additionally, as telesimulation becomes more common, manikins which address the specific technical challenges (remote monitor control, etc.) of telesimulation may become more available. 

## 6. Summary

Telemedicine has an increasing portfolio of applications that can be used to improve the safety and quality of neonatal resuscitation by providing a virtual connection from well-equipped regional perinatal centers to rural or otherwise underserved urban locations. Telemedicine offers a relatively low-cost and low-resource model of care to provide resuscitation education as well as direct resuscitation and procedural support, while maintaining the important health policy benefits of the regionalization of services. The opportunities telemedicine provides are beginning to be studied but there is certainly much more to learn about the effects of these interventions on patient outcomes, particularly in the neonate. Future research should focus on establishing standardized guidelines for the remote training of providers and investigating the outcomes of infants receiving care assisted, either directly or indirectly, by telemedicine.
